# Inducible Bronchus-Associated Lymphoid Tissues (iBALT) Serve as Sites of B Cell Selection and Maturation Following Influenza Infection in Mice

**DOI:** 10.3389/fimmu.2019.00611

**Published:** 2019-03-29

**Authors:** Hyon-Xhi Tan, Robyn Esterbauer, Hillary A. Vanderven, Jennifer A. Juno, Stephen J. Kent, Adam K. Wheatley

**Affiliations:** ^1^Department of Microbiology and Immunology, University of Melbourne, The Peter Doherty Institute for Infection and Immunity, Melbourne, VIC, Australia; ^2^Biomedicine, College of Public Health, Medical and Veterinary Sciences, James Cook University, Douglas, QLD, Australia; ^3^Melbourne Sexual Health Centre and Department of Infectious Diseases, Alfred Hospital and Central Clinical School, Monash University, Melbourne, VIC, Australia; ^4^ARC Centre for Excellence in Convergent Bio-Nano Science and Technology, University of Melbourne, Parkville, VIC, Australia

**Keywords:** germinal center (GC), influenza, iBALT, B cell, humoral immunity

## Abstract

Seasonally recurrent influenza virus infections are a significant cause of global morbidity and mortality. In murine models, primary influenza infection in the respiratory tract elicits potent humoral responses concentrated in the draining mediastinal lymph node and the spleen. In addition to immunity within secondary lymphoid organs (SLO), pulmonary infection is also associated with formation of ectopic inducible bronchus-associated tissues (iBALT) in the lung. These structures display a lymphoid organization, but their function and protective benefits remain unclear. Here we examined the phenotype, transcriptional profile and antigen specificity of B cell populations forming iBALT in influenza infected mice. We show that the cellular composition of iBALT was comparable to SLO, containing populations of follicular dendritic cells (FDC), T-follicular helper (Tfh) cells, and germinal center (GC)-like B cells with classical dark- and light-zone polarization. Transcriptional profiles of GC B cells in iBALT and SLO were conserved regardless of anatomical localization. The architecture of iBALT was pleiomorphic and less structurally defined than SLO. Nevertheless, we show that GC-like structures within iBALT serve as a distinct niche that independently support the maturation and selection of B cells primarily targeted against the influenza virus nucleoprotein. Our findings suggest that iBALT, which are positioned at the frontline of the lung mucosa, drive long-lived, and unique GC reactions that contribute to the diversity of the humoral response targeting influenza.

## Introduction

The generation of adaptive immune responses to infection requires the complex and elegant coordination of T and B lymphocytes, concentrated within secondary lymphoid organs (SLO), such as the spleen and lymph nodes. SLO are highly organized and regulated structures, facilitating antigen concentration, capture and presentation to naïve lymphocytes, thereby facilitating the activation, and differentiation of T and B cells into effector populations. Further specialized zones of germinal centers (GC) drive maturation of the humoral response via the selection of high affinity antibodies, providing an effective source of long-lived protection in the form of serum antibody and antigen-experienced memory B and T cell populations.

While SLO develop during embryogenesis at discrete anatomical locations (1), the induction of immunological structures resembling SLO has been reported at peripheral sites in response to inflammatory stimuli or infection with pathogenic microorganisms (2–5). Variably described as ectoptic lymphoid tissue or tertiary lymphoid organs, such lymphocyte aggregations also tend to form proximal to the bronchi within the lung and are termed inducible bronchus-associated lymphoid tissues (iBALT). Induction of iBALT has been extensively reported following pulmonary infections by bacterial (6, 7) or viral agents (8–10). iBALT can also form in response to exposure to particulates (11, 12) or pro-inflammatory mediators (13, 14), and chronic allergic or inflammatory diseases such as COPD and asthma (15). Despite conservation across numerous species and a wide number of contexts, the contribution of iBALT to humoral immunity against influenza is not fully understood.

While it is unlikely that a single pathway for iBALT genesis exists given the wide range of originating stimuli, the developmental models proposed for ectoptic lymphoid tissues have commonalities (16). Based on these models, cytokine and chemokine production from innate cells such as ILCs and γδT cells recruit pro-inflammatory granulocyte populations, which in turn drive the CXCL13-dependent recruitment of mature B cells into the lung (14, 17–19). Similar to the maintenance of conventional lymphoid tissues, T and B cells support the differentiation of stromal cells into mature follicular dendritic cells (FDC) and fibroblastic reticular cells (FRC), with homeostatic maintenance allowing these lymphoid-like structures to persist for months in the absence of ongoing inflammation or infection (20–22). While iBALT is decidedly pleiomorphic (16), several groups have reported the presence of B-cell clusters, networks of FDC, T cell areas, and zones resembling germinal centers, with iBALT supporting the proliferation of both B and T cells (8–10, 13, 23). Notably, iBALT can initiate protective humoral and T cell responses following influenza infection in mice lacking SLO (8, 9), although the relative importance of lung-generated responses in immunocompetent animals is unclear.

Using the influenza infection model in mice, we sought to address two questions. First, to what extent do the GC-like structures in the lung resemble GC in SLO? In particular, do iBALT GC-like structures facilitate B cell selection and affinity maturation? Second, to what extent is the lung a priming site for humoral immunity to pulmonary influenza infection in immunocompetent animals? We show GC responses in influenza infection-induced iBALT were comparable to GC in SLO in terms of cellular composition, phenotype, and transcriptional profile. However, the architecture of iBALT GC was non-classical, lacking discrete light and dark zone polarization and T cell compartmentalization to the light zone. Nevertheless, using labeled recombinant influenza antigens to probe B cell specificities, we find that influenza-specific GC responses within iBALT predominantly targeted the viral nucleoprotein, constituted a unique clonal repertoire relative to SLO, and demonstrated a comparable capacity to drive somatic mutagenesis and B cell selection requisite for affinity maturation. Our work has implications for the development of improved humoral immunity to influenza by establishing long-lived and unique GC reactions at the frontline of the lung mucosa.

## Materials and Methods

### Animal Infection

All animal procedures were approved by the University of Melbourne Animal Ethics Committee. C57BL/6 mice at 6–10 weeks of age were used. Mice were anesthetized by isoflurane inhalation prior to infection. For intranasal infections, mice were instilled with 50 μL volume of 50 TCID_50_ of A/Puerto Rico/8/34 (PR8).

### Confocal Microscopy

Fresh tissues were snap-frozen in O.C.T. compound (Sakura Finetek USA) and stored at −80°C. Tissues were sectioned at 7 μm thickness (Leica). Prior to staining, sectioned tissues were fixed in cold acetone solution (Sigma) for 10 min then rehydrated with PBS for 10 min and blocked with 5% (w/v) bovine serum albumin (Sigma) and 2% (v/v) normal goat serum (NGS). Cell staining was performed using the following antibodies: IgD (11-26c.2a; Biolegend), B220 (RA3-6B2; BD), GL7 (GL7; Biolegend), CD35 (8C12; BD), CD3 (17A2; Biolegend), CD169 (3D6.112; BD), CD86 (GL1, Biolegend), CXCR4 (2B11; BD), CD4 (GK1.5; Biolegend), Ki67 (11F6, Biolegend), BCL6 (K112-91; BD). To amplify the CXCR4-PE signal, tissues were stained sequentially with rabbit anti-PE antibody (polyclonal; Novus Biologicals) and a secondary goat anti-rabbit IgG Alexa Fluor 555 antibody (polyclonal; Life Technologies). Slides were sealed with ProLong Diamond Antifade Mountant (Life Technologies). Tiled Z-stack images covering were captured on a Zeiss LSM710 microscope. Post-processing of confocal images was performed with ImageJ v2.0.0.

### HA Proteins

Recombinant HA protein for use as flow cytometry probes was derived for A/Puerto Rico/08/1934 as previously described (24). Briefly, expression constructs were synthesized (GeneArt) and cloned into mammalian expression vectors. Proteins were expressed by transient transfection of Expi293 (Life Technologies) suspension cultures and purified by polyhistidine-tag affinity chromatography and gel filtration. Proteins were biotinylated using BirA (Avidity) and stored at −80°C. Prior to use, biotinylated HA proteins were labeled by the sequential addition of streptavidin (SA) conjugated to BV421, phycoerythrin (PE), or allophycocyanin (APC), and stored at 4°C. Recombinant influenza A H1N1 NP protein (11675-V08B; Sino Biological) was labeled with PE or APC fluorochromes using commercial conjugation kits, as per manufacturer's protocol (AB102918, AB 201807; Abcam).

### Flow Cytometry

For experiments requiring discrimination of blood and tissue populations, mice were intravenously labeled with 3 μg of CD45.2 antibody (104; eBioscience) in a 200 μL volume prior to tissue collection. Tissues were mechanically homogenized into single cell suspensions in RF10 media (RPMI 1640, 10% FCS, 1 × penicillin-streptomycin-glutamine; Life Technologies). Except for MLN, red blood cell lysis was performed with Pharm Lyse™ (BD). Isolated cells were Fc-blocked with a CD16/32 antibody (93; Biolegend). For all B cell experiments, cells were surface stained with Live/dead Aqua viability dye (Thermofisher) and B220 (RA3-6B2; BD), IgD (11-26c.2a; BD), CD45 (30-F11, BD), GL7 (GL7; Biolegend), CD38 (90; Biolegend), Streptavidin (BD), CD3 (145-2C11; Biolegend), and F4/80 (BM8; Biolegend). For LZ/DZ experiments cells were also stained with CD86 (GL1, Biolegend) and CXCR4 (2B11; BD). Antigen-specific B cells were detected based upon dual staining with HA or NP probes conjugated to APC and PE. For Tfh cell characterization, cells were stained in Transcription Factor Buffer Set (BD) with Live/dead Red viability dye (Life Technologies), BCL6 (IG191E/A8; Biolegend), CD3 (145-2C11; Biolegend), PD-1 (29F.1A12; Biolegend), CXCR5 (L138D7; Biolegend), CD4 (RM4-5; BD), B220 (RA3-6B2; BD), and F4/80 (T45-2342; BD). Stained cells were washed twice and fixed with 1% formaldehyde (Polysciences) for acquisition on a BD LSR Fortessa, or resuspended in Optimem (Life Technologies) for sorting on a BD FACSAria III.

### RNAseq and Data Analysis

Single cell suspensions were processed and stained as described above. Memory B cells (B220+ IgD- CD38hi GL7-), GC B cells (B220+ IgD- CD38lo GL7+), and T cells (CD3+) were sorted into RLT buffer (Qiagen) containing 0.14 M β-mercaptoethanol (Sigma). Post-sort, cells were suspended in a volume of RLT buffer representing 3.5x the sorting solution volume. Genomic DNA was removed using a gDNA eliminator (Qiagen), according the manufacturer's protocol. RNA was extracted by addition of 100% ethanol in a 5:7 volume ratio to flow-through solution and processed with the RNeasy Plus Micro Kit (Qiagen), according to the manufacturer's instructions. The Australian Genome Research Facility (Melbourne, Australia) performed the RNAseq with an Illumina HiSeq 2500 and obtained 100bp single reads. The library was prepared with a TruSeq Stranded mRNA Library Prep Kit (Illumina). RNAseq analysis was performed using the web-based Galaxy platform maintained by Melbourne Bioinformatics (25). Reads were mapped to the *Mus musculus* reference genome (mm10) using HISAT2 (26) and reads were quantified using HTSeq (27). Count matrices were generated and inputted into Degust (http://degust.erc.monash.edu) for data analysis and visualization with the Voom/Limma method selected for data processing. Heat maps were generated using Morpheus (The Broad Institute; https://software.broadinstitute.org/morpheus/). Raw sequence reads can be accessed with GEO code: (GSE124369).

### Sequencing and Analysis of Murine B Cell Receptor Genes

Sequencing of murine heavy chain immunoglobulin sequences was performed as previously described (28). Briefly, single B cells stained with the panel above and NP-PE or HA-BV421 probes were sorted into 96-well plates and cDNA prepared using SuperScript III Reverse Transcriptase (Life Technologies) and random hexamer primers (Life Technologies). Heavy chain immunoglobulin sequences were amplified by nested PCR using HotStar Taq polymerase (Qiagen) and multiplex primers binding V-gene leader sequences or the immunoglobulin constant regions. PCR products were Sanger sequenced (Macrogen) and VDJ recombination analyzed using High V-QUEST on IMGT (29). Clonality was determined based upon shared gene usage and CDR-H3 length and sequence similarity. Circular layout graphics were generated using the *Circlize* package in R (30).

### Statistics

Data are generally presented as the median ± interquartile range or the mean ± SD. Flow data was analyzed in FlowJo v9 (FlowJo) and all statistical analyses were performed using Prism v7 (GraphPad).

## Results

### Dynamics of iBALT Induction Following Intranasal Influenza Infection

**Figure 1:**
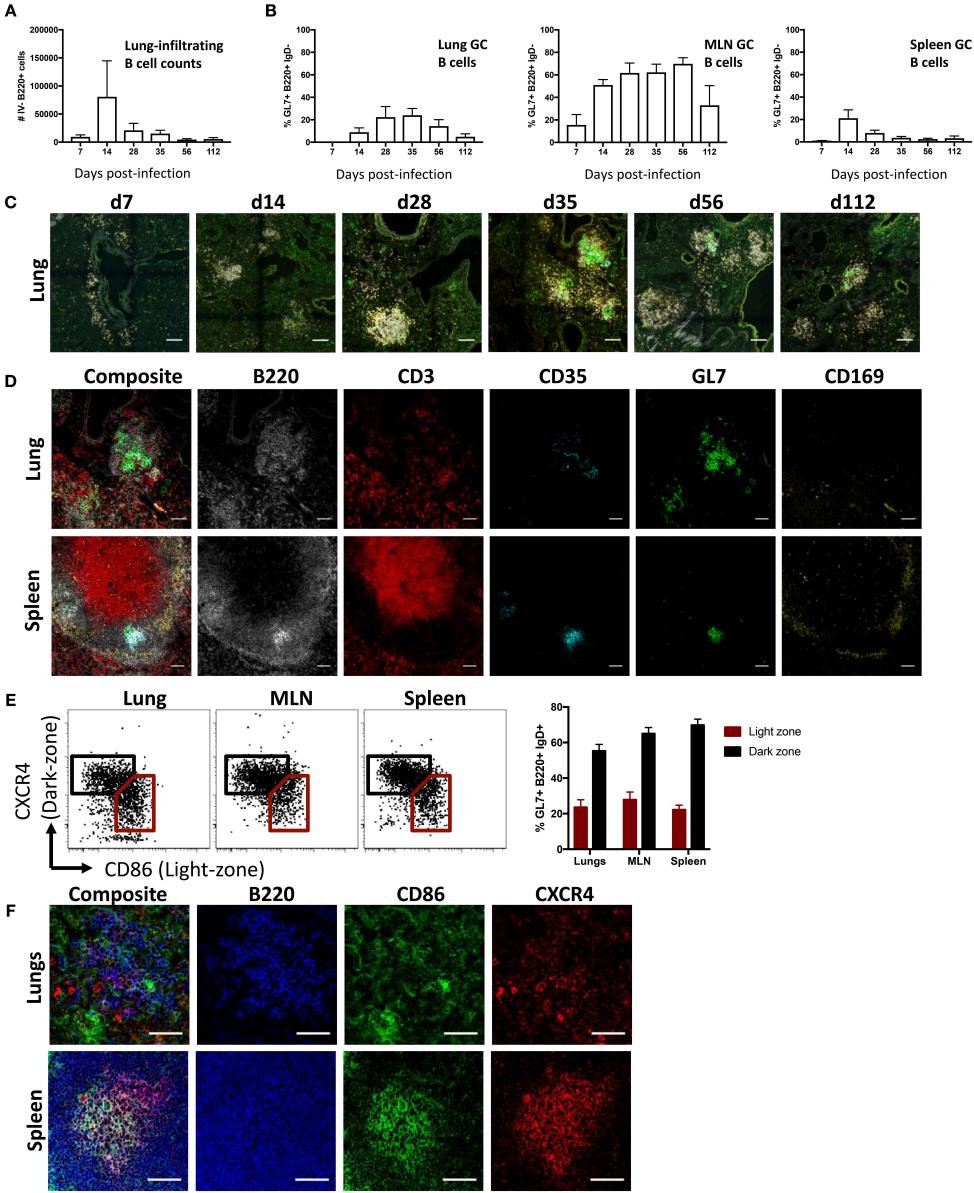
iBALT formation and characterization following intranasal influenza infection in mice. **(A)** Lung B cell infiltration (B220+ intravenous (IV) CD45.2-) and **(B)** frequency of GC B cells (B220+ IgD- CD38lo GL7+) across various tissues were measured in mice infected intranasally with A/Puerto Rico/08/1934. Data represent two independent experiment (*n* = 6). Error bars represent mean ± SD. **(C)** Induction and maturation of iBALT across various time-points visualized by composite images comprising B220 (orange), IgD (gray), GL7 (green), and CD35 (cyan); scale bar −100 μM. **(D)** GC cellular composition of lungs and spleen visualized by immunofluorescence staining at d35 post-infection; scale bar −50 μM. **(E)** Frequency and **(F)** visualization of light- and dark-zone GC B cells in lungs and SLO at d35 post-infection. Data represent a single experiment (*n* = 6). Error bars represent mean ± SD. Scale bar −50 μM.

To study lung B cell responses to influenza, we first performed intranasal infection of mice with A/Puerto Rico/08/1934 (PR8) virus. Consistent with previous reports (8, 10, 31, 32), intranasal infection resulted in a pronounced infiltration of B cells into the lung. Lung-infiltrating B cells, distinguished from blood-circulating populations by intravenous CD45.2 labeling, displayed peak numbers 14 days (d14) post-infection and persisted up to d112 (Figure 1A; gating Figure S1). A subpopulation of B cells expressing a GC-like phenotype (B220+ IgD- GL7+ CD38lo) were evident in lungs at d14 post-infection and detectable up to d112, albeit waning at the latter timepoint (Figure 1B). In comparison, mediastinal LN (MLN) displayed the largest proportion of GC B cells with continued maintenance at high frequencies up to d112 post-infection, in line with our previous observations (33). Splenic GC B cells frequencies peaked at d14, rapidly waned and was proportionally lowest amongst the tissues analyzed from d28 onward.

When visualized using confocal microscopy, lung tissues from infected mice similarly showed B cell infiltration and clustering (Figure 1C). Although B cell infiltration was observed at d7, cells were not structurally organized and were mostly dispersed around the bronchi regions. GC-like structures, demarcated by GL7 staining, were apparent at d14, peaking in size between d28 and d56, and subsequently waning in size by d112. When analyzed by flow cytometry, lung B cells gradually acquired a canonical GC phenotype in later timepoints, with full differentiation into GL7+ CD38lo and peak absolute numbers of GC B cells observed by d35 (Figure S2). As mature persistent GC responses are well-established in both iBALT and the draining MLN by d35, we selected this as a suitable timepoint for subsequent GC analysis. We contrasted the structural characteristics of putative lung GC with canonical splenic GC from infected mice at d35 post-infection (Figure 1D). Despite a pleiomorphic appearance (Figure S3), both FDC (CD35+) and highly clustered B cells (B220+) expressing GL7 were detected in iBALT analogous to the spleen. In contrast, T cells (CD3+) were dispersed throughout the lung tissues and CD169 staining, which demarcates subcapsular sinus macrophages in the spleen, was negligible in iBALT structures.

GL7 expression in SLO is tightly linked to GC localization (34). The distributed GL7 staining throughout iBALT led us to question if the observed GC-like B cell structures were functionally analogous to actual germinal centers. We therefore examined the differential expression CXCR4 and CD86, previously described for the delineation of dark- and light-zone resident cells in both human and murine GC (35). GC B cells from the lung displayed the characteristic differential expression profiles of CXCR4 and CD86, analogous to staining in MLN and spleen (Figure 1E). However, CXCR4 and CD86 staining visualized by immunofluorescence microscopy on lung resident B cell clusters was diffuse, lacking the discrete localization of light- and dark-zone staining seen in splenic samples (Figure 1F). Light and dark zone polarization requires differential chemokine expression by stromal cells, including FDC (36). Compared to the expected focused and GC-polarized FDC staining in MLN and spleen, FDC staining within the lung was diffuse with little indication of light-zone localization (Figure S4). Thus, while GC-like structures within influenza-induced iBALT display many phenotypic characteristics consistent with conventional GC within SLO, they appear morphologically unconventional.

### Transcriptional Profile of GC-Like B Cells From iBALT

**Figure 2:**
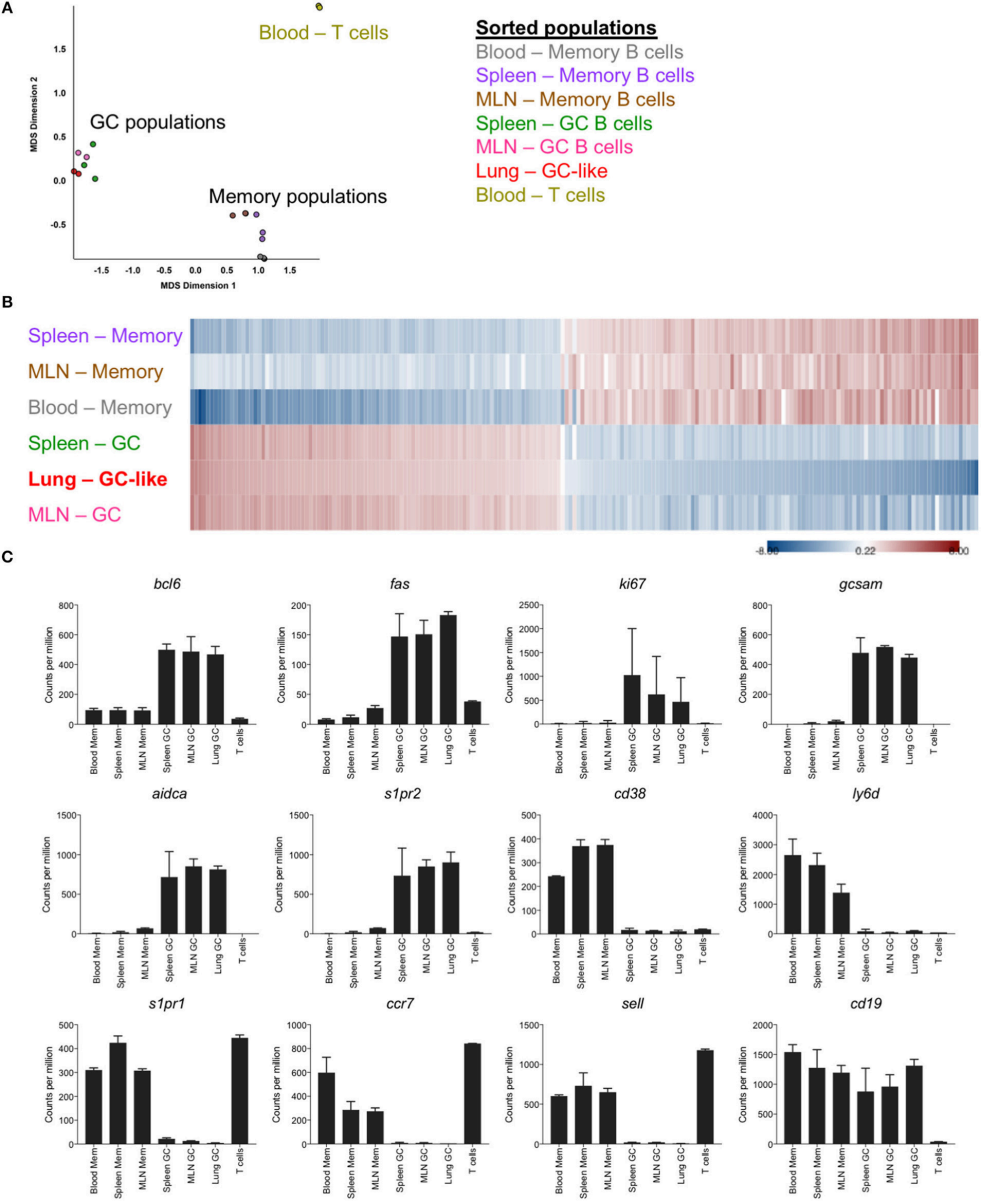
Transcriptional profile of GC-like B cells in iBALT. GC (B220+ IgD- GL7+ CD38lo) and memory B cells (B220+ IgD- GL7- CD38hi) were sorted from SLO and iBALT in mice at d35 post-infection with PR8. mRNA expression profiles were compared using RNAseq data from 2 to 3 independent sequencing experiments. **(A)** Multi-dimensional scaling plot showing clustering of memory and GC populations relative to T cell controls. **(B)** Heatmap of the top 200 differentially expressed genes, with shading indicating expression relative to the average (red–overexpressed, blue–underexpressed). **(C)** Differential expression of selected genes previously described to demarcate memory and GC subsets. Error bars represent mean ± SD.

To facilitate an in-depth comparison of GC B cells from different anatomical sites, memory and germinal center B cells were sorted from the lung, spleen, MLN and blood of mice at d35 post-infection (gating Figure S1), and differential gene expression profiles were generated by RNASeq. Based upon multi-dimensional scaling, iBALT GC-like B cells clustered closely with GC B cells from SLO and were distinct from both memory B cell populations and T cell controls (Figure 2A). This was similarly evident within a heatmap generated using the top 200 differentially expressed genes, where memory and GC-like populations displayed discrete expression profiles across lung-, MLN-, and spleen-localized cells (Figure 2B). Notably, iBALT GC-like B cells, but not memory B cells, expressed the master transcription factor *bcl6*, and canonical GC B cell markers *fas, aidca, gcsam*, and *s1pr2* in a manner comparable to GC B cells from SLO (Figure 2C). Conversely, we observed limited expression of genes associated with the memory phenotype in GC B cell populations (*ccr7, s1pr1, ly6d, cd38, sell*). Consistent with previous BrdU incorporation data (8), iBALT GC-like B cells appeared to be proliferating (expressing *ki67*).

### iBALT-Resident Tfh Cells Express Bcl6 and Ki67

**Figure 3:**
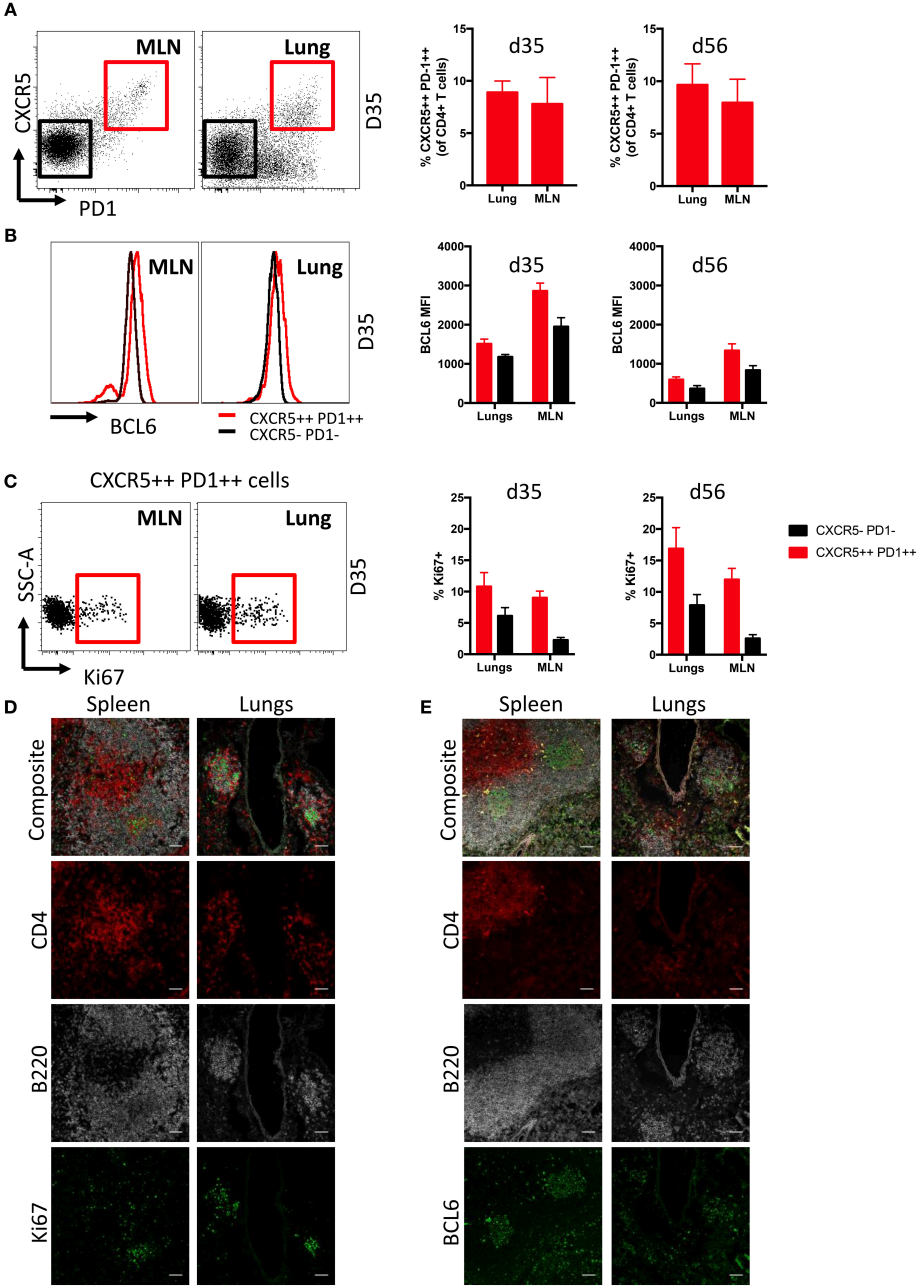
Characterization of Tfh cells in iBALT. **(A)** Frequency of Tfh cells (CD3+ CD4+ CXCR5++ PD1++) in MLN and lungs of mice at d35 and d56 post-infection with PR8. **(B)** Intracellular BCL6 staining measured by mean fluorescence intensities in Tfh and non-Tfh CD4 T cells (CD3+ CD4+ CXCR5- PD1-) isolated from MLN and lungs. **(C)** Frequency of proliferation marker Ki67 expressed in Tfh and non-Tfh CD4 T cells isolated from MLN and lungs. Data represent a single experiment (*n* = 4–5). Error bars represent mean ± SD. Visualization by immunofluorescence microscopy of **(D)** Ki67 and **(E)** BCL6 expression in T and B cells of spleen and lung iBALT; scale bar −50 μM.

Follicular T helper cells (Tfh) are critical for the generation and maintenance of germinal centers in SLO (36), but the presence of Tfh in lung iBALT induced by influenza infection is understudied. We found comparable frequencies of Tfh cells (CD3+ CD4+ CXCR5++ PD1++) were evident within the lungs and MLN of mice at d35 and d56 post-infection, encompassing close to 10% of the total CD4+ T cell population (Figure 3A; gating Figure S5A). Tfh from both MLN and lungs expressed elevated levels of the lineage-defining transcription factor BCL6 (37, 38) compared to conventional CD4+ T cells (CXCR5- PD1-) (Figure 3B), although with higher comparative expression (based upon fluorescence intensity) observed within MLN-derived Tfh cells. Intracellular Ki67 expression suggested that lung Tfh cells contained a similar proportion of recently proliferating cells compared to MLN at d35, but marginally higher proliferation than MLN Tfh cells at d56 (Figure 3C). B220+ cells expressing high levels of Ki67 and BCL6 were also present in both lungs and MLN of infected mice, although with frequencies higher in in the latter tissue (Figure S5B).

Ki67 (Figure 3D) and BCL6 (Figure 3E) expression in spleen and lungs of mice at d35 post-infection was visualized by confocal microscopy. As expected, the spleen showed distinct spatial segregation of T and B cell zones. In contrast, we found CD4 T cell and B cell clusters to be substantially smaller in the lungs and their spatial segregation was not clearly delineated. Mirroring expression measured by flow cytometry, Ki67 and BCL6 expression were detected in B220 and CD4 cells of both spleen and lungs, although expression of these markers was more evident in B220 cells. Overall, while we observed differences in anatomical localization, Tfh within iBALT-resident GC structures appear phenotypically analogous to SLO-resident Tfh cells.

### Specificity of GC-Resident B Cells for Major Influenza Antigens

**Figure 4:**
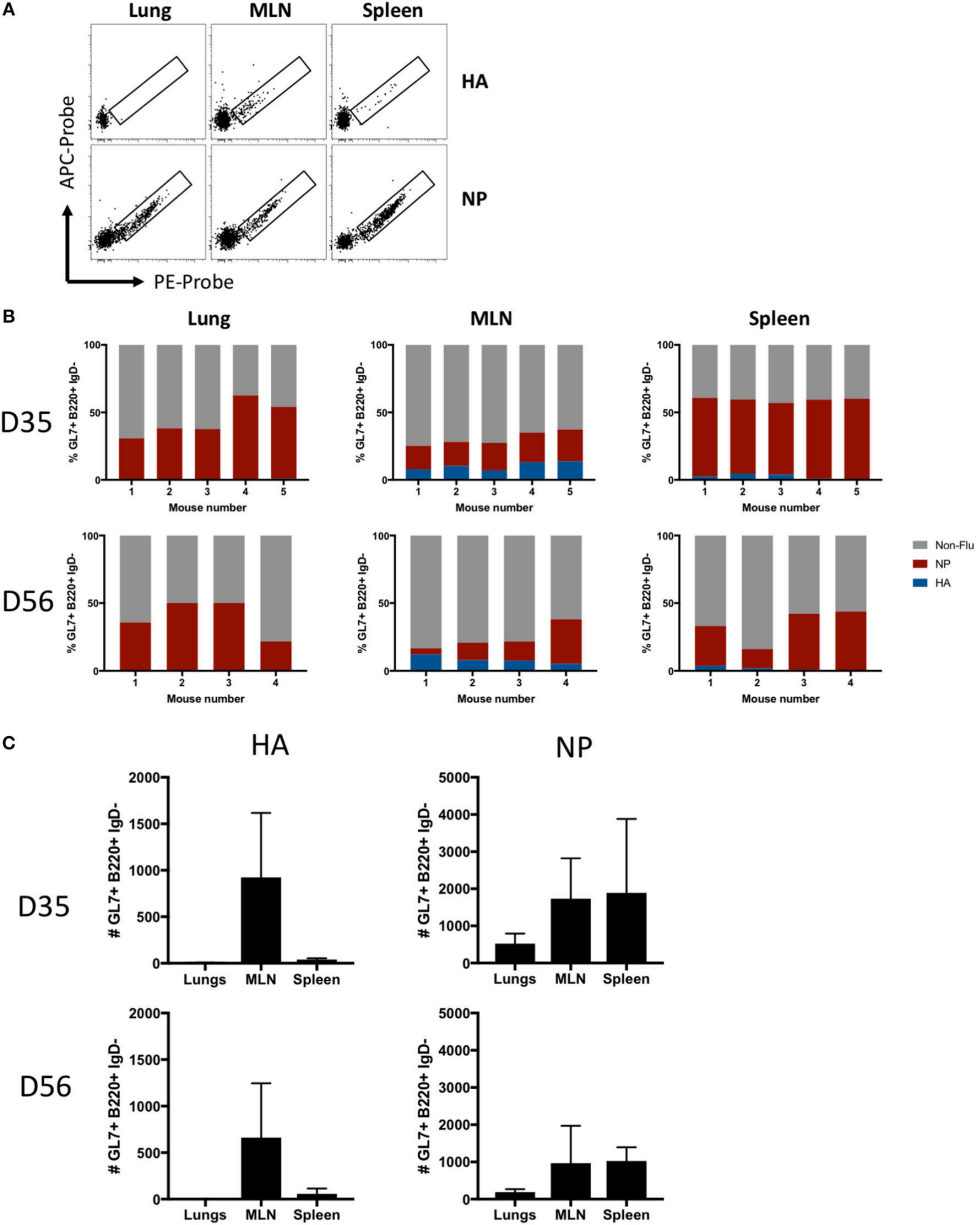
Antigen specificity of GC B cells in lung iBALT and SLO. **(A)** Representative HA and NP probe staining of GC B cells (B220+ IgD- CD38lo GL7+) isolated from lung iBALT, MLN, and spleen of mice at d35 post-infection with PR8. **(B)** Frequency of HA-, NP-specific, and undefined GC B cells isolated from individual mice at d35 and d56 post-infection. **(C)** Absolute counts of HA-specific and NP-specific GC B cells in lung iBALT, MLN, and spleen of mice at d35 and d56 post-infection. Data represent two independent experiment (*n* = 4–5). Error bars represent mean ± SD.

The antigen specificity of lung- or SLO-localized GC B cells was analyzed using recombinant influenza HA and NP probes (Figure 4A). NP-specific GC B cells were evident in all infected mice at both d35 and d56 post-infection at all sites, whereas HA-specific GC B cells were observed almost exclusively in the MLN and spleen, with little to no HA-specific GC B cells in the lungs (Figure 4B). In terms of absolute numbers of HA- and NP-specific GC B cells at both timepoints, the majority of HA-specific GC B cells were found in the MLN, a minor population in the spleen, and little to no HA-specific GC B cells observed in the lungs (Figure 4C). In contrast, NP-specific GC B cell were found at high frequencies and counts in all tissues, with MLN and spleen showing similar total cell counts while less cells were detected in lungs.

### GC Within iBALT Support Maturation of Influenza-Specific B Cells

**Figure 5:**
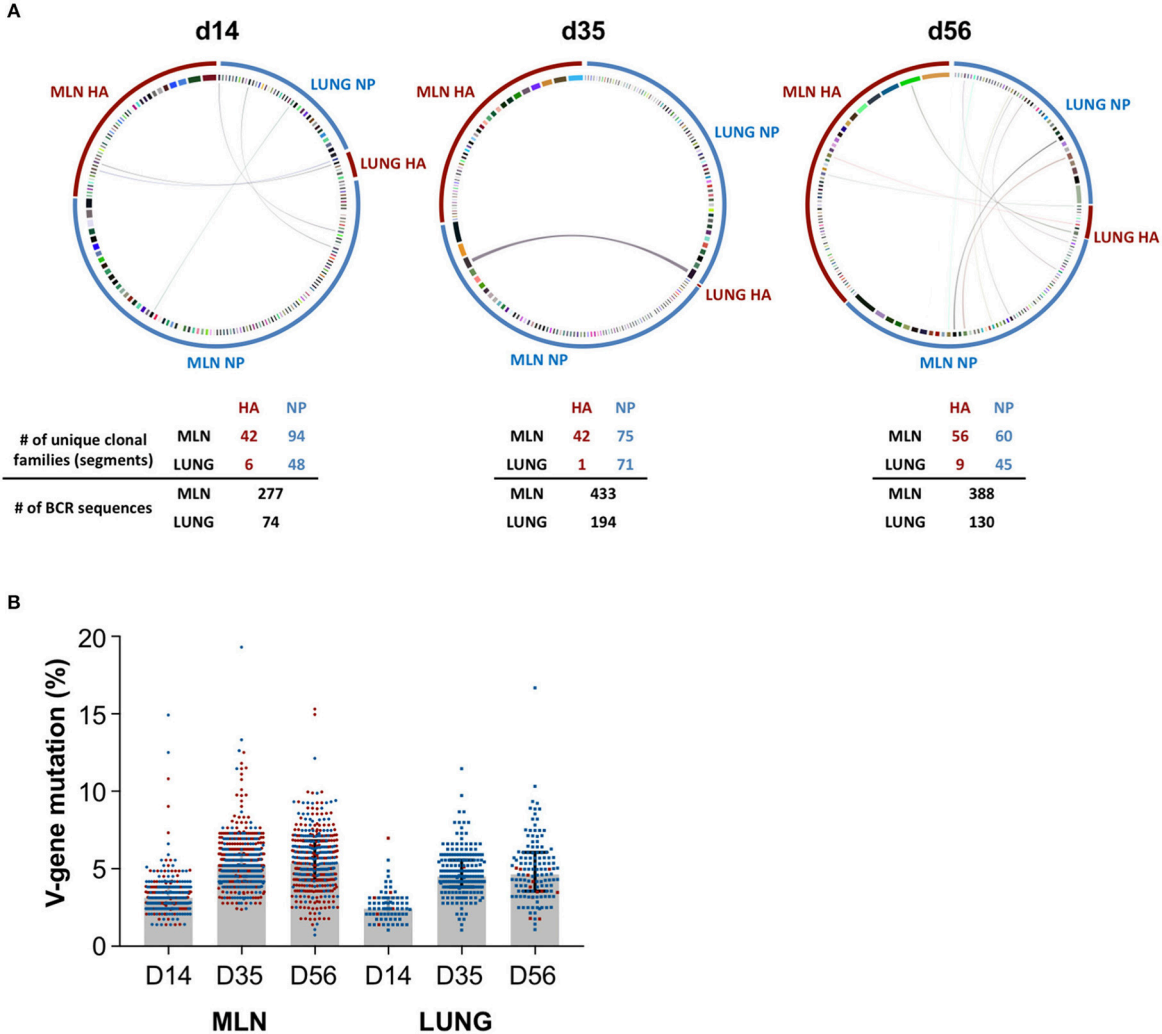
BCR sequencing of GC B cells in the lung and mediastinal lymph node. Heavy chain immunoglobulin sequences were recovered from NP- and HA-specific GC B cells sorted from the lung and mediastinal lymph nodes of PR8-infected mice on d14, d35, and d56. Data represents a single experiment with 5 mice per timepoint. **(A)** Circular plots detailing the clonal distribution of NP- and HA-specific populations from each site. Each segment represents a unique B cell clonotype, the width proportional to the number of clonal family members recovered. Shared clonotypes between sites are indicated by the linking arcs. Number of unique clonal families and BCR sequences are indicated below. **(B)** The degree of V-gene somatic mutation in recovered heavy chain immunoglobulin sequences from each site. NP- and HA-specific B cells are indicated in blue and red, respectively. Error bars represent median ± interquartile range.

We next examined if iBALT GC structures display a functional capacity to drive maturation of the influenza-specific B cell response. NP- and HA-specific B cell populations were sorted from GC in the lung and MLN at d14, d35, and d56 and heavy chain B cell receptor sequences were recovered, aligned and grouped into clonal families. Antigen-specific B cell populations in the lung (NP) and MLN (NP and HA) consisted of numerous clonally-expanded lineages (Figure 5A), indicating proliferation consistent with the observed Ki67 expression. We observed discrete compartmentalization of the humoral response, with minimal sharing of clonotypes and different clonal hierarchies seen between the lung and MLN, indicating B cell recruitment to each of these sites was highly segregated. This pattern was conserved across all animals and timepoints analyzed, and is consistent with previous reports that the lung hosts a unique B cell repertoire (39). The observed rates of V-gene somatic mutation in the MLN were comparable for both NP- and HA-specific GC B cells, increasing over time from a median 3.1% to 5.2% to 5.3% from d14 to d35 to d56 respectively (Figure 5B). Similarly within the lung, while few HA-specific B cells were recovered, somatic mutation in B cells that were mostly NP-specific increased from 2.4% to 4.5% to 4.6% across the same timepoints. Notably, the mutation load of influenza-specific GC B cells within iBALT remained consistently lower (~0.7%) in comparison to the MLN at each timepoint sampled. However, the rate at which somatic mutations accumulated was comparable to the MLN suggesting an equivalent capacity to drive maturation of humoral responses exists at both sites. Differences observed between the tissues are likely due to the delayed biogenesis of iBALT-localized GC.

## Discussion

There is considerable interest in the generation of local immunity within the lungs to protect from influenza and other respiratory pathogens. iBALT represent a focus of lymphoid tissues that has the potential to improve immunity within the lower respiratory tract. Despite the iBALT being pleiomorphic and less structurally defined than SLO GC structures, we found GC B cells in iBALT and SLO were transcriptionally similar and show that iBALT serve as a distinct anatomical niche for the maturation and selection of B cells primarily targeted against the influenza virus nucleoprotein.

The cellular composition of iBALT can vary, depending upon the stimuli used for induction (16). Consistent with previous reports, we observed cellular infiltration concentrated at bronchial junctions that included FDC (8), Tfh and conventional T cells (8, 9, 32), and germinal center B cell populations (10) following pulmonary influenza infection. In contrast, LPS-induced iBALT exhibits lung-infiltrating T cells with Tfh-like function but lacking classical CXCR5/BCL6 expression (13). Further, inoculation with LPS or whole *Pseudomonas aeruginosa* bacteria results in ectopic lung GC structures that lack FDC, in contrast to the CXCL13-expressing FDC observed following infection with modified vaccinia virus Ankara (7, 13, 40). Nuances in iBALT biogenesis appear attributable to the extent and nature of inflammation induced by infectious diseases compared to alternative pulmonary stimuli.

Germinal centers are highly specialized foci facilitating B cell proliferation, competition, and selective differentiation in order to drive high affinity serological antibody responses. We find that clusters of GC-like B cells observed within iBALT appear analogous to GC B cells in SLO with respect to cellular composition and phenotype. This includes evidence of proliferation, division into light- and dark-zone phenotypes, expression of canonical GC transcription factors, and the presence of Tfh cells. Here we extend previous studies (8, 40) by transcriptional profiling lung GC-like B cells by RNAseq. We found a conserved transcriptional signature was shared by GC B cells independent of anatomical location, with both lung- and SLO-resident populations expressing canonical transcription factors (e.g., *bcl6*), mediators of affinity maturation (e.g., *aidca*), and key chemotactic factors (e.g., *s1pr2*).

One key area of difference between GC within SLO vs. iBALT was the variant morphology, with a lack of highly distinct T and B cell zones observed in iBALT consistent with previous reports (8, 9, 41), and a lack of spatial segregation of B cells phenotypically characteristic of light- and dark-zone B cells. The impact of atypical GC architecture upon the development of B cell immunity in the lung is unclear. Previous studies have shown light- and dark-zone B cells exhibit largely overlapping gene expression patterns (42). Experiments using CXCR4-deficient mice (43) have established that B cells can cycle between these two states, and that discrete light- and dark-zone architecture is not a requisite for affinity maturation nor plasma cell differentiation (44). Based upon recovered BCR sequences from GC-resident B cells, we find GC in the lung maintain a capacity to drive maturation of the influenza-specific B cell response, with the continued accumulation of somatic mutations observed over 56 days post-infection. While overall mutation frequencies were consistently higher in the draining MLN, the rate of accumulation was comparable between the two sites suggesting no detectable defect in the functionality of iBALT-localized GC.

Given the importance of anti-HA antibody for protection against influenza (45), the contribution of iBALT to serological HA-specific antibody seems minimal. Certainly, non-neutralizing antibody responses, such as those targeting NP, can contribute to immunity via Fc-mediated functions or antibody-dependent cellular cytotoxicity, and further provide protection against heterosubtypic influenza virus challenge in mice (46–50). iBALT might conceivably act as a source of mucosal-resident antibody-secreting cells (ASCs) that contribute to ongoing barrier protection in the lung. We observed strong compartmentalization of B cell receptor repertoires within anatomical sites, implying that alternative B cell clonotypes are recruited to and maintained in lung GC compared to SLO. This additional clonotypic diversity may be advantageous during recurrent exposure to antigenically diverse pathogens such as influenza. The extended persistence of iBALT, reported for up to 3 months (9, 10, 31, 40), may also serve as a broader niche to position B cells proximal to a site of recent mucosal barrier damage. Interestingly, we find a significant proportion of lung-resident GL7+ B cells, comprising up to half of total cells, were of undefined specificity. This corroborates previous studies demonstrating that the iBALT can act as a general priming site, thus supporting the proliferation of GC B cells binding antigens unrelated to the primary inducing agent (23, 51). While these GC B cells may also simply represent B cells specific for alternative antigenic targets of the influenza virus not captured by our probes (52), or non-specific bystander B cells adopting a GC phenotype, they may also be elicited by antigenic translocation of microorganisms as a result of lung damage during infection. Additional definition of the specificities of iBALT-resident GC B cells is warranted.

In summary we find GC-like structures in iBALT are phenotypically, transcriptionally and functionally comparable to those within SLO, yet recruit a distinct subset of B cell clonotypes into the humoral immune response. While iBALT can drive B cell diversification and may facilitate residence of B and T lymphocytes proximal to the mucosa, the protective value of iBALT within immunocompetent hosts remains unclear, and in the context of our influenza infection model, seems largely restricted to targeting the viral nucleoprotein.

## Data Availability

The RNAseq datasets generated for this study can be found in Gene Expression Omnibus, NCBI (Accession GSE124369).

## Author Contributions

H-XT, SK, and AW designed the study. H-XT, RE, HV, JJ, and AW performed the experiments. H-XT, RE, JJ, SK, and AW wrote the manuscript. All authors read and revised the manuscript.

### Conflict of Interest Statement

The authors declare that the research was conducted in the absence of any commercial or financial relationships that could be construed as a potential conflict of interest.
