# Supplementary figures and images for: Inducible Bronchus-Associated Lymphoid Tissues (iBALT) Serve as Sites of B Cell Selection and Maturation Following Influenza Infection in Mice
Source: Front Immunol. 2019 Mar 29;10:611. doi: 10.3389/fimmu.2019.00611 (PMC6450362; doi:10.3389/fimmu.2019.00611)

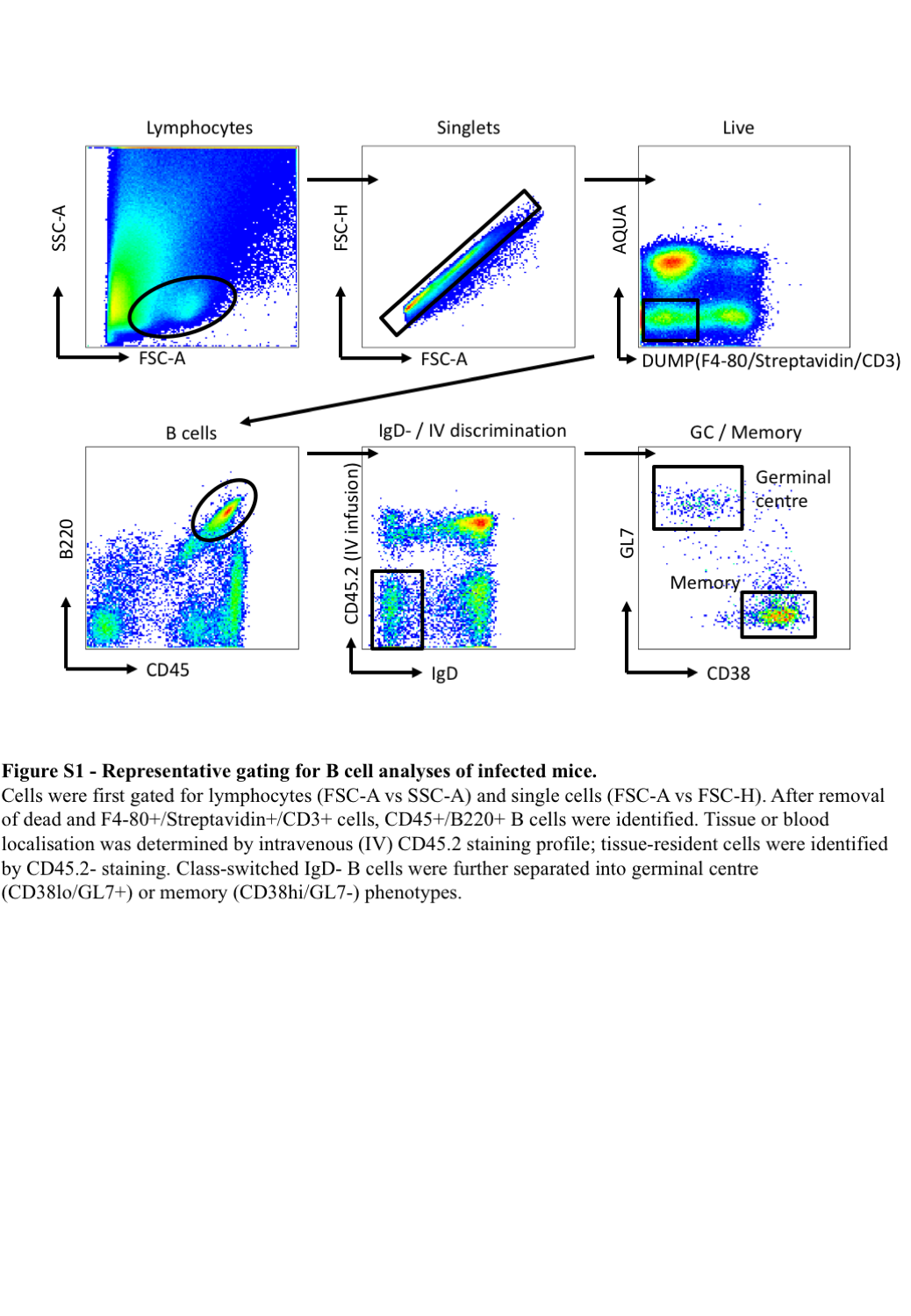

Supplement: Supplementary file 1 [file Image_1.tiff]

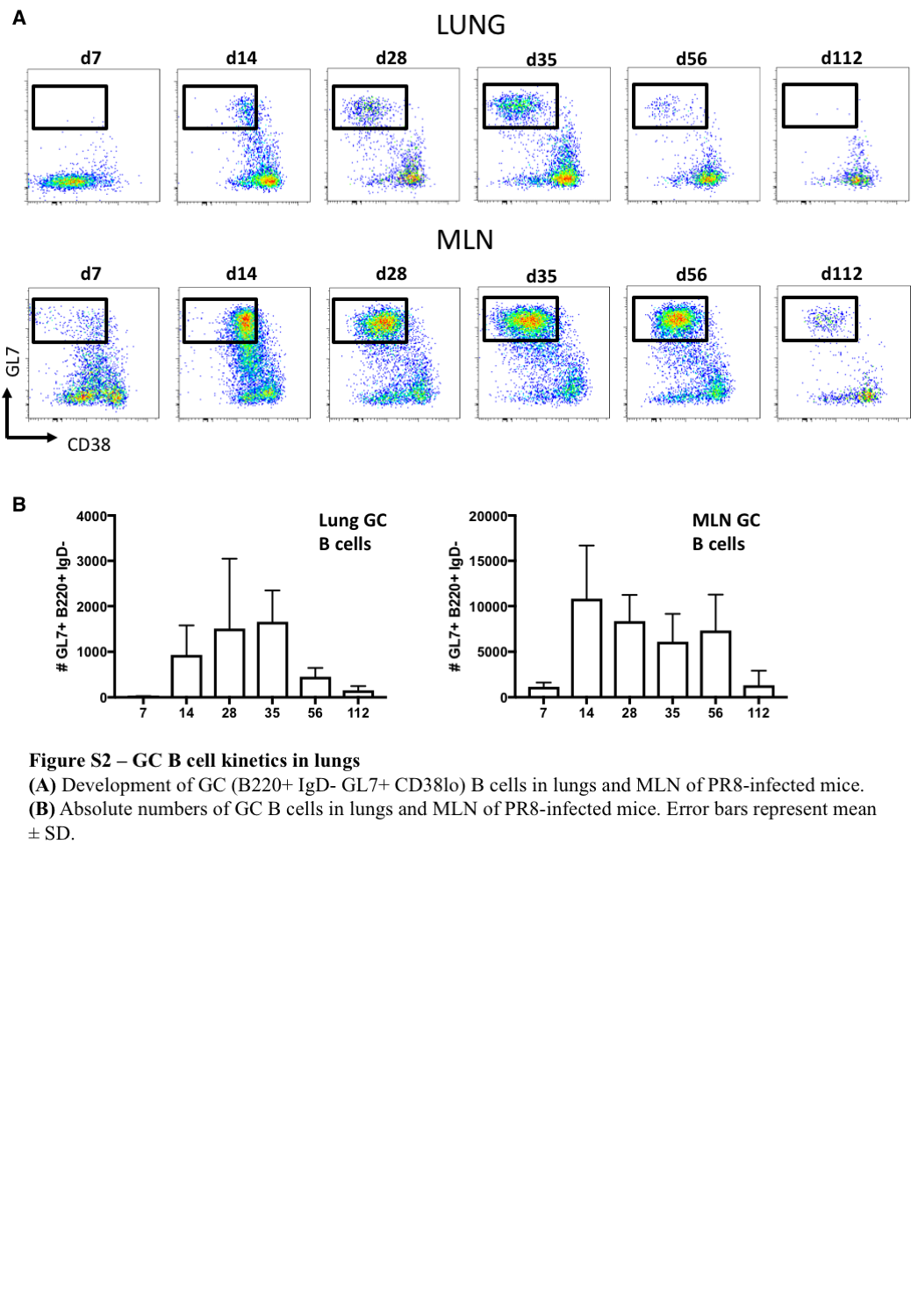

Supplement: Supplementary file 2 [file Image_2.tiff]

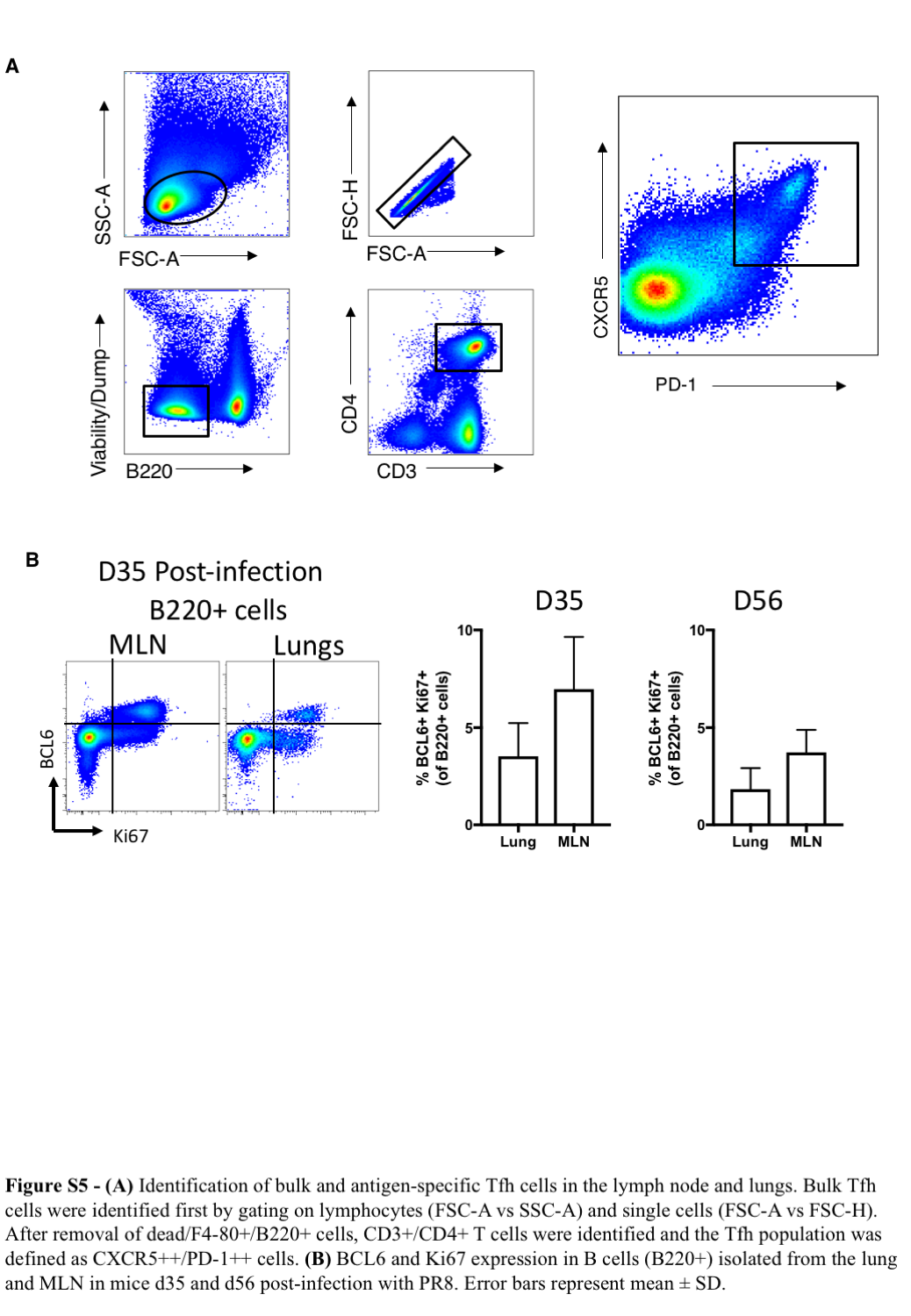

Supplement: Supplementary file 5 [file Image_5.tiff]
