# Supplementary material for: Inducible Bronchus-Associated Lymphoid Tissues (iBALT) Serve as Sites of B Cell Selection and Maturation Following Influenza Infection in Mice
Source: Front Immunol. 2019 Mar 29;10:611. doi: 10.3389/fimmu.2019.00611 (PMC6450362; doi:10.3389/fimmu.2019.00611)
Supplement: Supplementary file 3 [file Image_3.pdf]

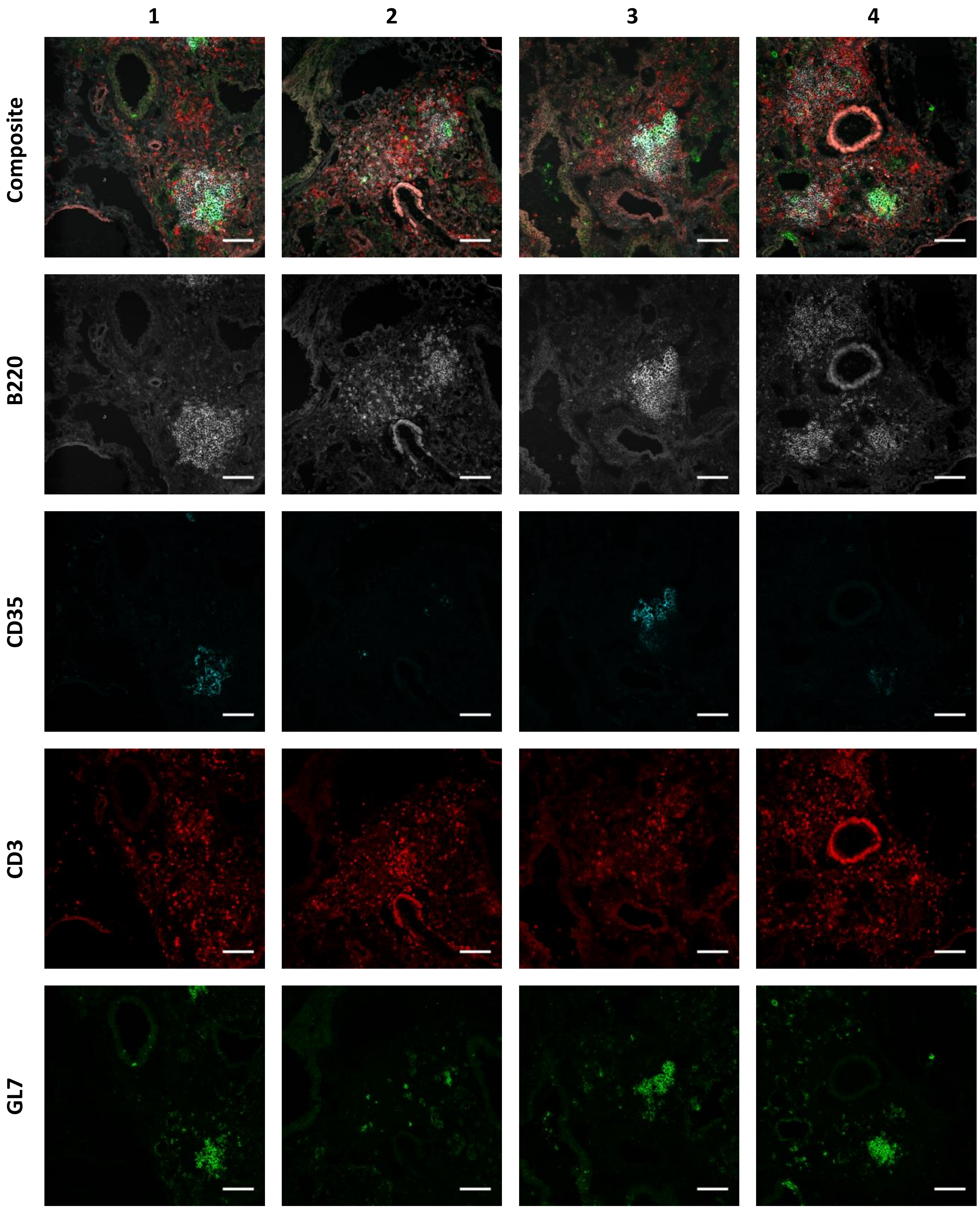

Figure S3 - Pleiomorphic iBALT structures seen in the lung at d35 post-infection with PR8. Lung tissues were stained with B220 (grey), CD35 (cyan), CD3 (red) and GL7 (green); scale bar – 100 µM.
