# Supplementary material for: Inducible Bronchus-Associated Lymphoid Tissues (iBALT) Serve as Sites of B Cell Selection and Maturation Following Influenza Infection in Mice
Source: Front Immunol. 2019 Mar 29;10:611. doi: 10.3389/fimmu.2019.00611 (PMC6450362; doi:10.3389/fimmu.2019.00611)
Supplement: Supplementary file 4 [file Image_4.pdf]

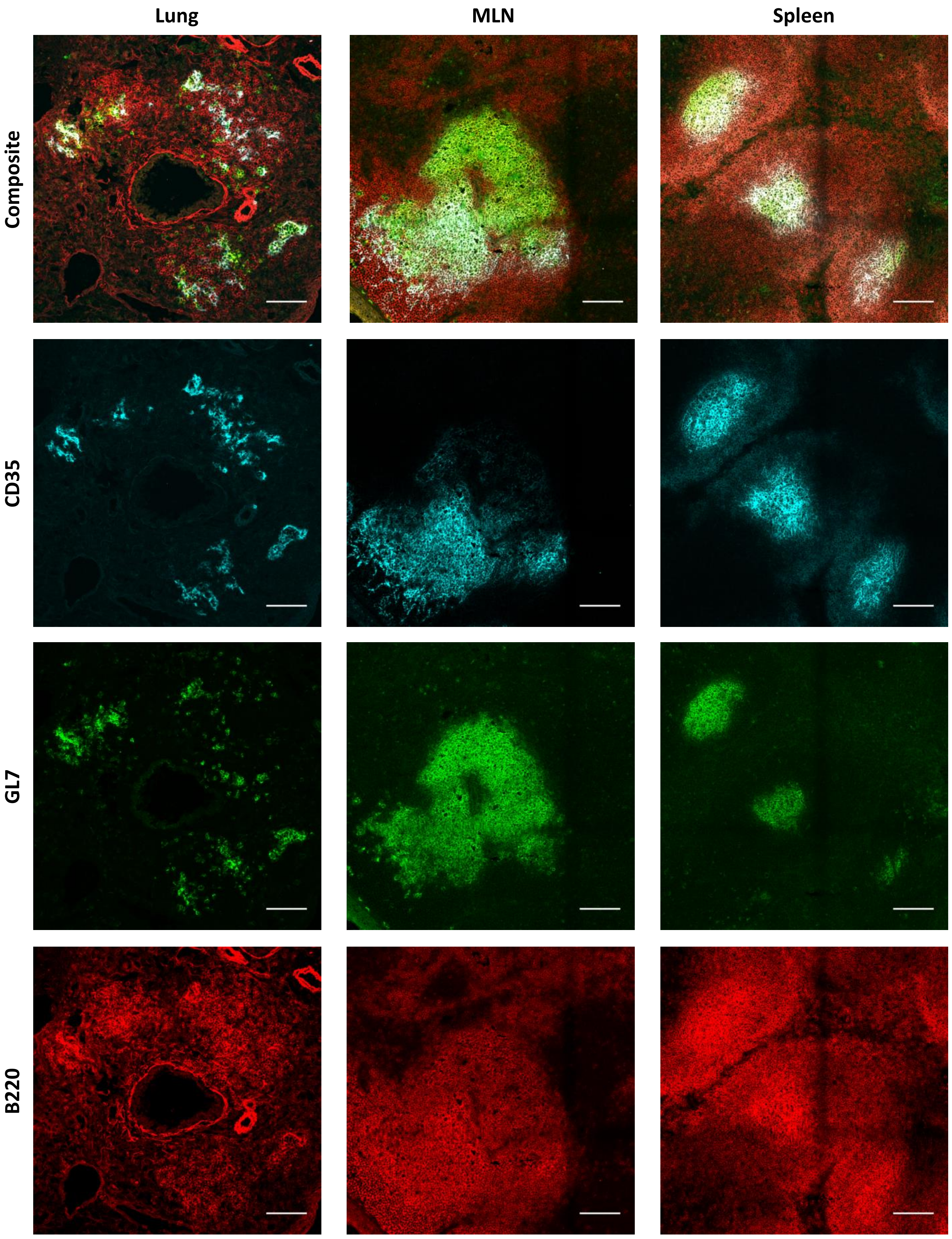

Figure S4 – Comparison of germinal centres of lungs, spleen and MLN from PR8-infected mice. Tissues were stained with CD35 (cyan), GL7 (green) and B220 (red); scale bar – 100 µM.
